# Association of Depression with Inflammation in Hospitalized Patients of Myocardial Infarction

**Published:** 2014

**Authors:** Yu-Xiu Shang, Wen-Qing Ding, Hong-Yan Qiu, Feng-Ping Zhu, Shu-Zhen Yan, Xue-Liang Wang

**Affiliations:** 1 Yu-Xiu Shang, Department of Epidemiology and Biostatistics, School of Medicine, Xi’an Jiaotong University, Xi’an, Shaanxi, China; 2 Wen-Qing Ding, Department of Public Health, Ningxia Medical University, Yinchuan, Ningxia, China.; 3 Hong-Yan Qiu, Department of Public Health, Ningxia Medical University, Yinchuan, Ningxia, China.; 4 Feng-Ping Zhu, Department of Public Health, Ningxia Medical University, Yinchuan, Ningxia, China.; 5 Shu-Zhen Yan, Department of Public Health, Ningxia Medical University, Yinchuan, Ningxia, China.; 6 Xue-Liang Wang, Department of Epidemiology and Biostatistics, School of Medicine, Xi’an Jiaotong University, Xi’an, Shaanxi, China

**Keywords:** Myocardial infarction, Inflammation, Depressive symptoms

## Abstract

***Objective:*** The aim of this study was to examine the associations between depression and inflammatory markers in patients admitted to the hospital for myocardial infarction.

***Methods:*** Inflammatory cytokines, including high-sensitivity C-reactive protein (hs-CRP), interleukin (IL)-1β, IL-6, and tumor necrosis factor-α (TNF-α) were assessed in a group of 75 depressed participants (score of ≥ 12) and compared to a control group of 75 nondepressed participants (score < 12), all who had been admitted to the hospital for myocardial infarction. The presence of depressive symptoms was assessed using the Beck Depressive Symptoms Inventory II Scale (BDI-II).

***Results:*** Depressed myocardial infarction participants had significantly greater levels of TNF-α (*t* = 2.070, *P *< 0.05) compared with control myocardial infarction participants. The BDI-II score was positively correlated with TNF-α levels (*r* = 0.222, *P < *0.05).

***Conclusions:*** These results indicate that the presence of depressive symptoms is positively associated with TNF-α levels among patients who have suffered from myocardial infarction.

## INTRODUCTION

The presence of depressive symptoms is not only a prognostic factor in patients with established coronary heart disease (CHD) but is also a risk factor for the incidence of cardiovascular disease in healthy individuals.^1^^,^^2^ However, the underlying physiological mechanisms are not well understood. Several physiological mechanisms have been proposed, including changes in coagulation factors, genetic factors that increase the risk of having both depressive symptoms and CHD, and alterations in glucose metabolism. Inflammation is a frequently proposed physiological mechanism that links the presence of depressive symptoms to the development and progression of CHD.^3^

CHD is a chronic inflammatory process that involves an immunological response to vascular endothelial cell injury. In patients with CHD, increased inflammatory risk markers have been associated with increased cardiovascular events. Inflammatory risk markers that have been evaluated include C-reactive protein (CRP), interleukin (IL)-6, and tumor necrosis factor-α (TNF-α).^4^^,^^5^ Prospective epidemiological studies have found that increased levels of these particular inflammatory cytokines are predictive of future CHD in still healthy populations.^6^^,^^7^ In addition, studies of depressed patients with or without CHD have found that the presence of depressive symptoms is associated with higher levels of the inflammatory cytokines CRP, IL-6, IL-1, and TNF-α.^8^^-^^10^ Additionally, antidepressant treatment has been shown to decrease the levels of several of these inflammatory factors.^11^^,^^12^ Furthermore, patients with depressive symptoms show circulating patterns of immune activation similar to those seen in CHD.

Research performed in the past decade has increasingly demonstrated that inflammatory processes play a central role in the pathogenesis of atherosclerosis.^13^ The presence of depressive symptoms can increase the risk of CHD by inducing or promoting inflammatory processes. However, several studies have described contradictory results, and no significant differences in inflammatory cytokines were found when depressed and nondepressed otherwise healthy individuals were compared.^14^^,^^15^ These data indicate that there is an inconsistent link between the presence of depressive symptoms and inflammatory cytokine levels. The purpose of this study was to explore the relationship between depressive symptoms and inflammatory cytokines in patients who had suffered from myocardial infarction.

## METHODS

***Subjects:*** A cross-sectional study was conducted in three hospitals in the Ningxia Hui Autonomous Region. All patients were enrolled by cardiologists at three hospitals: the General Hospital of Ningxia Medical University, the People’s Hospital of Ningxia Hui Autonomous Region, and the Zhongwei City People’s Hospital. Institutional review board approval was gained from the Ethics Committee of Ningxia Medical University, and all patients gave informed written consent. Patients were recruited from consecutive admissions during hospitalization for myocardial infarction between April 2008 and April 2009.

Myocardial infarction was diagnosed if the patient met at least two of the following three criteria of the World Health Organization and Joint European Society of Cardiology/American College of Cardiology Committee for the Redefinition of myocardial infarction: typical chest pain, characteristic electrocardiographic changes, and a serial rise in serum creatine phosphokinase levels.

Patients were excluded if they had any of the following characteristics: 1) chronic infection or other inflammatory disease (15 exclusions), 2) suffered from a significant psychiatric illness other than depressive symptoms and received antidepressants or other psychotropics, 3) previous history of myocardial infarction (8 exclusions), 4) were too medically unstable to complete the interview (24 exclusions), or 5) were greater than 80 years of age (3 exclusions). A total of 50 patients were excluded. A total of 280 patients were approached, and 202 (72%) gave informed consent and completed baseline interviews, at which time blood samples were collected. The major reason for exclusion was patient refusal to permit a blood sample to be drawn. A total of 150 patients were enrolled in the study, while the patients were still hospitalized for the myocardial infarction. Of these, 75 met the diagnostic criteria for depressive symptoms, and the remaining 75 served as controls. Patients were subsequently interviewed by trained cardiologists and nurses in the step-down unit, who used a standard study questionnaire. The study questionnaire included social and demographic variables (age, sex, marital status, education, employment status, and financial status) and risk factors of myocardial infarction (hypertension, diabetes, cholesterol levels, body mass index (BMI), smoking, and physical exercise frequency). A BMI ≥ 28 was considered obese. Data on hypertension, diabetes mellitus, and cholesterol levels were recorded from medical records. The patients completed the Beck depressive symptoms Inventory-II (BDI-II). A BDI-II score ≥ 12 was required to classify a patient as depressed. Blood samples were drawn within 72 h of symptom onset, while the patients were still in the hospital.

***Assessment of*** ***depressive symptoms*** ***:*** Depressive symptoms were assessed using the BDI-II. The BDI-II is a 21-item self-reported questionnaire to measure the presence and severity of depressive symptoms according to the presence of symptoms for at least two weeks. Items assess symptoms that correspond to criteria for diagnosing depressive symptoms from the Diagnostic and Statistical Manual of Mental Disorders, 4th edition (DSM-IV)*.* Each item is scored on a 4-point scale ranging from 0 to 3. The total score ranges from 0 to 63, with higher scores indicating greater levels of depressive symptoms. Scores ≤ 4 indicate no depressive symptoms, scores of 5–13 indicate mild depressive symptoms, scores of 14–20 indicate moderate depressive symptoms, and scores ≥ 21 indicate severe depressive symptoms. The BDI-II has been validated as a sensitive, specific, and predictive tool for measuring depressive symptoms. The cut-off chosen as a lower threshold for detecting depressive symptoms was a BDI score of 12 points, which was based on the findings of a study by Frasure-Smith;^16^ therefore, a patient with a BDI score of ≥ 10 is at increased risk of cardiac mortality during the first 18 months after hospital discharge. A score of 10 or more on the BDI-I is equivalent to a score of 12 or more on the BDI-II. 

***Inflammatory marker measurements:*** Blood samples were drawn from the antecubital vein between 8:00 and 9:00 am, with the participant in a seated position after fasting for 12 h and abstaining from alcohol. Serum was divided into 1.5 ml aliquots and stored at -70°C until analysis. High-sensitivity CRP (hs-CRP) was measured by immunonephelometry (Dade Behring). The IL-1β, IL-6, and TNF-α levels were measured using ELISAs (R&D Systems) according to the manufacturer’s instructions. The inflammatory assays were carried out in the Central Laboratory of the General Hospital of Ningxia Medical University.

***Statistical analysis*** ***:*** The data are presented as mean ± standard deviation (SD). Differences in baseline characteristics between depressed and control participants were evaluated by the Student’s t*-*test for continuous and normally distributed variables or by the chi-squared (χ^2^) test for discrete variables. Because the inflammatory cytokine results showed skewed distributions, we analyzed the data after log transformation. Analysis of covariance was used to adjust for known risk factors of myocardial infarction, including age, sex, smoking status, alcohol consumption, BMI, physical activity, systolic blood pressure, and diabetes mellitus. Pearson correlation coefficients between log-transformed inflammatory cytokine levels, depressive symptoms score, and cardiovascular risk factors were computed. Analysis of variance was used to assess the relationship between log-transformed inflammatory cytokine levels.

Due to technical problems leading to blood hemolysis, data on serum CRP levels were not available for two participants, IL-1β for four participants, IL-6 for eight participants, and TNF-α for eleven participants. Therefore, the analysis did not include these samples. Statistical significance was defined as a two-tailed *P *< 0.05. All statistical analyses were performed using SPSS 11.5 for Windows.

## RESULTS

The study group included 150 myocardial infarction patients (77 females and 73 males) aged 46–79 years old, with a mean age of 62.8 ± 10.1 years old. Of the 77 females, 62.7% were depressed, while only 37.3% of the 73 males were depressed (*P *< 0.05). The basic characteristics of all case and matched control participants are presented in Table-I. Depressed and control participants were very similar with respect to demographic characteristics and cardiovascular risk factors (*P= not significant *for all variables except two). The two exceptions were education level and marital status, in which depressed participants had significantly lower educational levels than controls (*t *= -4.693, *P *< 0.001), and depressed participants were less likely to be married than controls (*χ*^2^ = 4.246, *P *< 0.05). Depressed patients had significantly higher depressive symptom scores than controls (*t* = 14.749, *P *< 0.001).

The mean values after log transformation of inflammatory cytokine levels of the two groups are presented in Table-II. Participants with depressive symptoms had significantly higher levels of TNF-α (*t* = 2.070, *P *< 0.05) compared with control participants. In contrast, CRP, IL-1β, and IL-6 levels were not different between the depressed and control participants. Due to the fact that several factors may confound relationships between the BDI score and inflammatory cytokines, analysis of covariance were performed. TNF-α was independently associated with the BDI-II score, even after adjustments were made for several confounders (*P < *0.05).

Pearson correlation coefficients between inflammatory cytokines, cardiovascular risk factors, and the BDI-II score are presented in Table-III. The BDI-II score was positively correlated with TNF-α levels (*r* = 0.222, *P < *0.05), but unrelated to CRP, IL-1β, and IL-6 levels. CRP positively correlated with BMI (*r* = 0.204, *P < *0.05) and age (*r* = 0.372, *P < *0.01). IL-1β was negatively correlated with total cholesterol (*r* = -0.309, *P < *0.05).

## DISCUSSION

The purpose of this study was to test the hypothesis that depressive symptoms in patients who had suffered from myocardial infarction were associated with inflammation, as indicated by elevated inflammatory marker levels. In line with previous reports, our results demonstrated that patients with depressive symptoms had significantly higher levels of TNF-α in comparison to the controls, after adjustments for potential confounders were made. These results indicate that depressive symptoms in patients with CHD are significantly associated with inflammation.

**Table-I:** Patient Characteristics

	**Control patients** **(** **n = ** **75)**	**Patients with depressive symptoms ** **(n = 75)**	**t/** **χ** ^2^	P
Age, years	61.8 ± 11.3	63.6 ± 8.9	0.976	0.331
Educational level, years of school	9.4 ± 3.2	7.3 ± 2.1	-4.693	<0.001
Married, n (%)	71 (95%)	63 (84%)	4.246	0.039
Current or former smoker, n (%)	29 (39%)	23 (31%)	0.969	0.325
Body mass index, kg/m^2^	25.1 ± 4.9	23.7 ± 5.8	-1.566	0.120
Fasting blood glucose, mmol/L	6.00 ± 1.66	6.04 ± 2.50	0.113	0.910
Postprandial blood glucose, mmol/L	7.90 ± 2.48	7.76 ± 2.78	-0.307	0.759
Systolic blood pressure, mmHg	131 ± 22	132 ± 24	0.282	0.778
Diastolic blood pressure, mmHg	84 ± 16	81 ± 14	-0.882	0.380
Total cholesterol, mmol/L	4.94 ± 1.42	4.43 ± 1.13	-2.381	0.019
Total triglyceride, mmol/L	2.77 ± 1.91	2.29 ± 1.36	-1.769	0.079
HDL cholesterol, mmol/L	1.13 ± 0.41	1.06 ± 0.36	-1.116	0.266
LDL cholesterol, mmol/L	2.30 ± 1.04	2.26 ± 0.88	-0.290	0.772
Depressive symptom scores	6 ± 4	22 ± 9	14.749	<0.001

Values are presented as the mean ± SD; P-values were obtained by the Student’s t-test for all variables except for marital and smoking status, which were analyzed by the χ^2^　test

**Table-II:** Differences in inflammatory cytokines between control patients and patients with depressive symptoms

**Variable**	**Group**	**n**	**Mean ** **± ** **SD**	**t**	**P** * **(unadjusted)**	**P** ** **(CHD-risk adjusted)**
Hs-CRP, mg/L	Controls	73	-0.14 ± 0.58	0.059	0.953	0.186
	Depressed	75	-0.15 ± 0.60			
IL-1β, pg/mL	Controls	73	1.74 ± 0.08	1.286	0.202	0.077
	Depressed	73	1.76 ± 0.05			
IL-6, pg/mL	Controls	71	0.43 ± 0.25	0.506	0.614	0.214
	Depressed	71	0.45 ± 0.23			
TNF-α, pg/mL	Controls	68	-0.24 ± 0.28	2.070	0.042	0.046
	Depressed	71	-0.35 ± 0.17			

* P values were obtained by the Student’s t*-*test after the data were log transformed;

** analysis of covariance, adjusted for age, sex, smoking status, alcohol consumption, body mass index (BMI), physical activity, systolic blood pressure, and diabetes mellitus.

**Table-III:** Pearson correlation coefficients for relationships among inflammatory cytokines, depressive symptoms score, and CHD risk factors

**Variable**	**Hs-CRP**	**IL-1** **β**	**IL-6**	**TNF-α**	**Depressive symptoms score**
Age	0.372**	0.032	-0.040	0.001	0.278**
BMI	0.204*	0.088	0.116	-0.046	-0.123*
SBP	-0.111	-0.043	0.004	0.079	0.008
Total cholesterol	0.005	-0.309*	0.098	-0.185	-0.066
HDL cholesterol	-0.052	0.069	0.031	-0.053	0.017
Depressive symptoms score	0.021	0.131	-0.082	0.222^*^	…

* P < 0.05;

** P < 0.01

Whether inflammation leads to depressive symptoms or whether depressive symptoms lead to inflammation has not been revealed. Indeed, the cause of the association between depressive symptoms and inflammation is largely unknown, and the relationship may be reciprocal using a positive feedback mechanism. Depressive symptoms are accompanied not only by mental anguish but also by alterations in fundamental processes, including endocrine signaling pathways for growth and reproduction, and the activity of both the hypothalamic-pituitary-adrenal axis and sympathetic nervous system.^17^ Depressive symptoms may promote a mild inflammatory response by triggering dysregulation of hormonal systems (the hypothalamic-pituitary-adrenal and sympathetic-adrenal-medullary axes) and increasing susceptibility to infections that are associated with atherosclerosis.^18^ Over time, this inflammatory response contributes to myocardial infarction by facilitating atherosclerotic plaque instability, which precipitates the rupture or fissuring of the existing plaque and the formation of occlusive thrombi. This cascade of events could explain the increased morbidity and mortality rates seen in myocardial infarction patients with depressive symptoms.

Alternatively, the synthesis of inflammatory cytokines could provoke neuroendocrine changes that are interpreted by the brain as stressors. This in turn would induce hyperactivity of the hypothalamic-pituitary-adrenal axis to produce hormonal products that might lead to depressive symptoms.^19^

In this study, we found no significant differences in the levels of CRP, IL-1β, or IL-6 between the depressed and the non-depressed groups of patients. Previous studies have found elevated CRP, IL-1β, and IL-6 levels in patients with depressive symptoms compared to healthy participants.^20^ The variable results, to some extent, may be due to the intrinsic variable characteristics of the depressive symptoms, recent infectious diseases, and prior medication use. Our study only documents the inconsistency of these observations.

***Limitations: ***This study had several limitations. First, we used self-reported questionnaires for assessing depression rather than a diagnostic interview with a psychologist. Self-reported questionnaires may overestimate the prevalence of depression compared with a diagnostic interview. Therefore, the proportion of depression may have been overestimated in our study. Second, although we tried to exclude people with concurrent infections, a small percentage of subjects may have had subclinical infections. The presence of an infection may influence the levels of the inflammatory markers investigated.

## CONCLUSION

The presence of depressive symptoms in patients who had suffered from myocardial infarction was associated with increased TNF-α levels. Therefore, increased TNF-α levels may be associated with depressive symptoms.

## Notes

***Author Contributions:*** YXS conceived and designed the study, performed statistical analysis, and wrote the manuscript. WQD, YXS, FPZ, and SZY performed data collection and analysis. XLW reviewed and edited the manuscript. YXS takes responsibility and accountability for all aspects of the work in ensuring that questions related to the accuracy or integrity of any part of the work are appropriately investigated and resolved. All authors approve the final version of this manuscript.

## Notes

***Source of funding: ***This study was supported by grants from Ningxia Natural Science Foundation (NZ0882) and Ningxia Colleges and Universities Technology Research Project (Ning Jiao Gao (2008) No. 293).
